# Supplementary material for: Analysis of Cultivable Microbial Community during Kimchi Fermentation Using MALDI-TOF MS
Source: Foods. 2021 May 12;10(5):1068. doi: 10.3390/foods10051068 (PMC8151656; doi:10.3390/foods10051068)
Supplement: Supplementary file 1 [file foods-10-01068-s001.zip › foods-1198978-supplementary.pdf]

Supplementary Materials

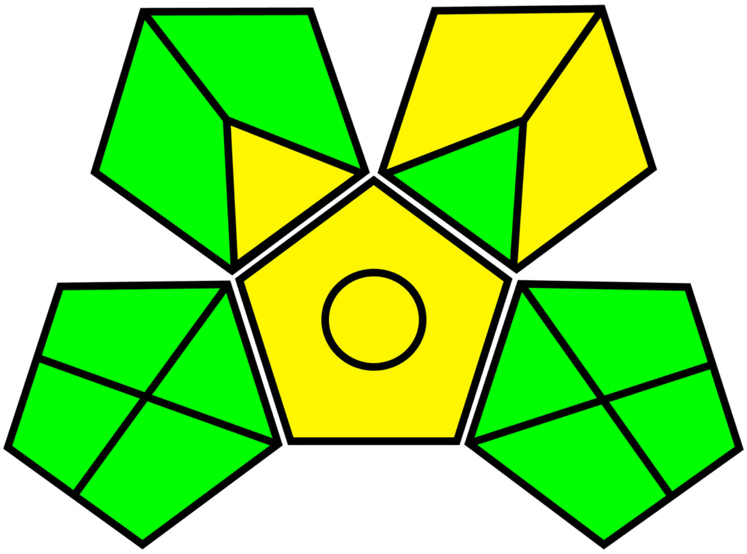

Figure S1. Greenness assessment profile for proposed MALDI-TOF MS method for investigating the microbial community in kimchi by GAPI tool

Table S1. The green analytical procedure index of the proposed MALDI-TOF MS method

Parameters	MALDI-TOF
Sample preparation	
Collection (1)	*In-line*
Preservation (2)	None
Transport (3)	None
Storage (4)	None
Type of method: direct or indirect (5)	Simple procedures
Scale of extraction (6)	Micro-extraction
Solvents/reagents used (7)	Green solvent
Additional treatments (8)	None
Reagents and solvents	
Amount (9)	2 µL per sample
Health hazard (10)	Formic acid (NFPA[1]: 3), Acetonitrile (NFPA: 2)<br>Trifluoroacetic acid (NFPA: 3), NaOH (NFPA: 3)<br>Distilled water (NFPA: 0)
Safety hazard (11)	Formic acid (NFPA: 0), Acetonitrile (NFPA: 2)<br>Trifluoroacetic acid (NFPA: 0), NaOH (NFPA: 1)<br>Distilled water (NFPA: 0)
Instrumentation	
Energy (12)	≤ 0.1 kWh per sample
Occupational hazard (13)	Hermetic sealing of analytical process
Waste (14)	0 mL
Waste treatment (15)	None

[1]NFPA: National Fire Protection Association
